# ROS and ERK1/2-mediated caspase-9 activation increases XAF1 expression in dexamethasone-induced apoptosis of EBV-transformed B cells

**DOI:** 10.3892/ijo.2013.1949

**Published:** 2013-05-20

**Authors:** GA BIN PARK, YUNOCK CHOI, YEONG SEOK KIM, HYUN-KYUNG LEE, DAEJIN KIM, DAE YOUNG HUR

**Affiliations:** 1 Department of Anatomy and Research Center for Tumor Immunology, Inje University College of Medicine, Busan 614-735, Republic of Korea; 2 Department of Internal Medicine, Inje University Busan Paik Hospital, Busan 614-735, Republic of Korea

**Keywords:** Epstein-Barr virus, B cell, dexamethasone, reactive oxygen species, ERK1/2, X-linked inhibitor of apoptosis-associated factor 1, Puma, Bax, apoptosis

## Abstract

Dexamethasone (Dex) inhibits the growth of diverse types of cancer cells and is utilized clinically for the therapy of hematological malignancies. In this study, we investigated the molecular mechanisms of Dex action in the apoptosis of Epstein-Barr virus (EBV)-transformed B cells. We showed that Dex inhibited the proliferation of EBV-transformed B cells and induced apoptosis by activating caspase-9, -3 and -8. While activation of caspase-9 was triggered as early as 2 h after Dex treatment, cleavage of caspase-8 was deferred and was found 8 h after the exposure. Dex-dependent activation of caspase-8 was blocked by the specific caspase-9 inhibitor, z-LEHD-fmk. Moreover, Dex significantly increased the expression of X-linked inhibitor of apoptosis (XIAP)-associated factor 1 (XAF1) and induced the translocation of XAF1 into the cytosol. Cytosolic XAF1 with Puma induced the translocation of Bax into mitochondria. Dex led to up-regulation of reactive oxygen species (ROS) generation and the phosphorylation of ERK1/2 after the exposure. We speculated that ROS generation might be the first event of Dex-induced apoptosis because ROS inhibitor NAC abrogated ROS production and ERK1/2 activation, but PD98059 did not block ROS production. NAC and PD98059 also suppressed the translocation of XAF1, Puma and Bax into mitochondria. These results demonstrated that Dex-mediated activation of caspase-9 via ROS generation and ERK1/2 pathway activation resulted in the activation of caspase-8 and the increment of XAF1, thereby induced apoptosis of EBV-transformed B cells. These findings suggest that Dex constitutes a probable therapy for EBV-associated hematological malignancies.

## Introduction

Epstein-Barr virus (EBV) is a human gamma-herpesvirus, infecting over 90% of the adult human population (1). EBV, as one of the most common viruses, is the causative factor of infectious mononucleosis and strongly involved with the development of various human malignant diseases, including Hodgkin’s lymphoma, Burkitt’s lymphoma, post transplant lymphoproliferative disorders, nasopharyngeal carcinoma, immunoblastic B lymphoma associated with HIV, some gastric carcinomas, and autoimmune diseases such as multiple sclerosis, Sjögren’s syndrome and rheumatoid arthritis (2). However, the precise role of EBV in the pathogenesis of these diseases is not yet clear.

Glucocorticoids (GCs) are known to regulate cell proliferation, differentiation, development and inflammation. Dexamethasone (Dex), a synthetic GCs, prevents the cell growth of many hematologic malignant cells and solid tumor cell types, including multiple myeloma (3), leukemia (4), prostate cancer (5), hepatoma (6), melanoma (7), osteosarcoma (8), lung cancer (9), breast cancer (10) and ovarian cancer cells (11). However, the biological effect and molecular events leading from Dex treatment in EBV-transformed B cells are still not understood completely.

XIAP-associated factor 1 (XAF1) is a nuclear protein and a binding partner that directly interacts with endogenous XIAP (X-linked inhibitor of apoptosis), resulting in the redistribution of XIAP from the cytoplasm to the nucleus for sequestration, thereby antagonizing the anti-caspase activity of XIAP (12). XAF1 was not only an apoptosis-promoting factor, but is also involved in the cellular stress response (13). XAF1 is expressed ubiquitously in all normal cells, in contrast to extremely low or undetectable levels in several cancer cells (14). Likewise, deficiency of XAF1 expression is strongly associated with tumor progression. Overexpression of XAF1 enhances chemosensitivity and cell death, and inhibits tumor growth in various cancers including gastric, colon, pancreatic and prostate cancers (15–18). Although XAF1 is thought to be a pro-apoptotic nuclear protein, after the re-localization of XAF1 to mitochondria, it promotes translocation of Bax into mitochondria and cytochrome *c* release from mitochondria (19). However, the role of XAF1 in apoptosis of EBV-transformed B cells and its putative correlation with the reactive oxygen species (ROS) and ERK1/2 pathway have not been studied.

In this study, we aimed to study the effect of XAF1 on cellular response to Dex in EBV-transformed B cells and the underlying molecular mechanisms. We were interested in whether ERK1/2 would have any regulatory role in other apoptotic pathways, such as the XAF1 signaling pathway, upon Dex treatment. We report a study on Dex-induced apoptosis in EBV-transformed B cells demonstrating that caspase-9 activation and XAF1 expression are induced by ROS production and ERK1/2 activation and mediate both in the induction of apoptosis and in translocation of Bax into mitochondria.

## Materials and methods

### Preparation of stock of EBV virions and generation of EBV-transformed B cells

Cell-free EBV virions were prepared from culture supernatant of B95-8 marmoset cell line. To establish EBV infection of B cells from normal peripheral blood mononuclear cells (PBMCs), PBMCs were isolated from peripheral blood of a healthy donor by Ficoll-Paque (Amersham Life Science, Buckinghamshire, UK) gradient centrifugation. PBMCs were added to EBV virions stock in a culture flask, and after 2-h incubation at 37°C, RPMI-1640 culture medium (HyClone) and 1 mg/ml of cyclosporine A (Sigma-Aldrich, St. Louis, MO, USA) were added to cells (1×10^6^ cells/ml). The cultures were incubated for 2–4 weeks. This study was approved by the Institutional Bioethics Review Board at the Medical College of Inje University, and all donors gave informed consent for the study.

### Proliferation measurement by AlamarBlue

Cells (5×10^4^ cells/well) were cultured in medium containing 10% FBS in 96-well plates. After 24 h, cell proliferation was measured by AlamarBlue (Serotec Ltd, Kidlington, UK) assay. AlamarBlue was added (10% by volume) to each well and relative fluorescence was determined 9 h later by SpectraMax M2e Multi-Detection Microplate Reader (Molecular Devices, Sunnyvale, CA; excitation, 530 nm; emission, 590 nm). Relative fluorescence unit (RFU) values were expressed as mean ± SEM of three determinations.

### Quantification of apoptotic cells by flow cytometry

The level of Dex-induced apoptosis in human EBV-transformed B cells (4 weeks, 5×10^5^ cells/ml) and normal PBMCs was measured by flow cytometry using FITC-labeled Annexin V and 7-AAD (BD Biosciences, San Diego, CA, USA). To decide optimal conditions, experiments were performed using variable concentrations (0, 10, 50, 100 and 200 *μ*M) and variable durations of incubation (2, 4, 8, 16 and 24 h). To investigate the effects of caspase inhibitors, cells were pretreated with z-LEHD-fmk (z-Leu-Glu(OMe)-His-Asp-(OMe)-fluoremethylketone, 20 *μ*M, a caspase-9 inhibitor; Calbiochem, San Diego, CA, USA) for 2 h before Dex treatment. To inhibit generation of ROS or ERK1/2 cascade, cells were pretreated with NAC (N-acetylcysteine, 10 mM, antioxidant; Sigma-Aldrich) or PD98059 (10 *μ*M, Calbiochem) for 1 h. Cells were then harvested, washed in PBS, and incubated with Annexin V and 7-AAD in binding buffer at room temperature for 15 min in the dark. The stained cells were analyzed using a FACSCalibur flow cytometer (BD Biosciences) equipped with CellQuest Pro software (BD Biosciences).

### Detection of mitochondria membrane potential (Δψ_m_) and intracellular reactive oxygen species (ROS) generation

The changes in mitochondrial membrane potential (Δψ_m_) were determined using DiOC_6_ (3,3’-dihexyloxacarboxyanine iodide; Molecular Probes, Eugene, OR, USA). Cells were treated with methanol (MetOH) or Dex for 24 h, harvested, washed twice in PBS, resuspended in PBS supplemented with DiOC_6_ (20 nM), incubated at 37°C for 15 min in the dark, and immediately analyzed by flow cytometry. The intracellular accumulation of ROS was examined by flow cytometry after being stained with the fluorescent probe, DCFH-DA (10 *μ*M, 2′,7′-dichlorodihydrofluorescein diacetate; Molecular Probes). DCFH-DA was deacetylated in cells by esterase to a non-fluorescent compound, DCFH, which remains trapped within the cell and is cleaved and oxidized by ROS in the presence of endogenous peroxidase to a highly fluorescent compound, DCF (2′,7′-dichlorofluorescein). EBV-transformed B cells were seeded in 6-well plates (5×10^5^ cells/ml), treated with or without Dex, and incubated with 10 *μ*M DCFH-DA for 30 min at 37°C. Then cells were washed, resuspended in PBS, and ROS levels were determined using a FACSCalibur flow cytometer (BD Biosciences).

### Reverse transcription polymerase chain reaction

Total RNA was isolated using an RNeasy mini kit (Qiagen, Hilden, Germany). RNA was transcribed into cDNA using oligo(dT) primers (Bioneer, Daejeon, Korea) and reverse transcriptase. To investigate apoptosis-associated molecules, PCR amplification was performed using specific primer sets (Bioneer) for XAF1 (upstream primer, 5′-TTCAGCTCCTGAAAGGGAAA; downstream primer, 5′-TTCAGCAGCTTGACTTGGAA), XIAP (upstream primer, 5′-GTGCCACGCAGTCTACAAATT CTGG; downstream primer, 5′-CGTGCTTCATAATCTGCCA TGGATGG), Bax (upstream primer, 5′-CCAAGAAGCTGAG CGAGTGT; downstream primer, 5′-CAGCCCATGATGGTT CTGAT), Noxa (upstream primer, 5′-AGGACTGTTCGTGTT CAGCTC; downstream primer, 5′-GTGCACCTCCTGAG AAAACTC), and Puma (upstream primer, 5′-GTGTAGAGG AGACAGGAATC; downstream primer, 5′-GCTCGTACTGT GCGTTGAGG). A specific primer set for β-actin (upstream primer, 5′-ATCCACGAAACTACCTTCAA; downstream primer, 5′-ATCCACACGGAGTACTTGC) was used as a control and PCR was performed using Prime Taq Premix (GeNet Bio, Chungnam, Korea). PCR products were analyzed by agarose gel electrophoresis and visualized with ethidium bromide under UV light using the multiple Gel DOC system (Fujifilm, Tokyo, Japan). Data were analyzed using ImageJ 1.38 software (National Institutes of Health, Bethesda, MD). Experiments were performed in triplicate.

### Western blot analysis

After treatment, cells were harvested and lysed in RIPA buffer (Elpis Biotech, Daejeon, Korea) containing a protease inhibitor cocktail (AEBSF, aprotinin, Bestatin hydrochloride, E-64, EDTA and leupeptin hemisulfate salt; Sigma-Aldrich). To address phosphorylation events, an additional set of phosphatase inhibitors (Cocktail II, sodium orthovanadate, sodium molybdata, sodium tartrate and imidasole; Sigma-Aldrich) was added to the RIPA buffer (Elpis Biotech, Daejeon, Korea). Protein concentration was determined using a BCA assay kit (Pierce, Rockford, IL). Proteins (10 *μ*g/sample) were immediately heated for 5 min at 100°C. Total cell lysates (5×10^6^ cells/sample) were subjected to SDS-PAGE on gel containing 15% (w/v) acrylamide under reducing conditions. Separated proteins were transferred to nitrocellulose membranes (Millipore Corp., Billerica, MA, USA), and then the membranes were blocked with 5% skim milk and commercial western blot analysis was performed. Chemiluminescence was detected using and ECL kit (Advansta Corp., Menlo Park, CA, USA) and the multiple Gel DOC system (Fujifilm). The following primary Abs were used: caspase-8, caspase-3, caspase-9, β-actin, Bax, Puma, Noxa, XAF1, phospho-JNK (Thr^183^/Tyr^185^), JNK, phospho-p38 MAPK (Thr^180^/Tyr^182^), p38 MAPK, phospho-ERK1/2 (Thr^202^/Tyr^204^), and ERK1/2 (Cell Signaling Technology, Beverly, MA, USA); Ref-1 and COX-IV (Santa Cruz Biotechnology, Santa Cruz, CA, USA); and β-tubulin (BD Biosciences). Data were analyzed using ImageJ 1.38 software.

### Measurement of XAF1, Bax and Puma translocation

Following treatment, mitochondrial and cytosol cellular fractions were prepared using a Cytosol/Mitochondria Fractionation kit (Calbiochem). Cells (1×10^7^) with or without various treatments were harvested by centrifugation at 600 × g for 5 min at 4°C and washed twice with cold PBS. Afterward, the cells were resuspended in 250 *μ*l Cytosol Extraction buffer containing a protease inhibitor cocktail and 1 mM dithiothreitol (DTT). After incubation on ice for 10 min, cells were homogenized on ice using a dounce tissue homogenizer. Homogenized cells were centrifuged at 700 × g for 10 min at 4°C, and supernatants were collected. Supernatants were then centrifuged again at 10,000 × g for 30 min at 4°C. The resulting supernatants were harvested and designated as cytosolic fractions and the pellets were resuspended in 50 *μ*l Mitochondria Extraction buffer containing a protease inhibitor cocktail and 1 mM DTT and designated as mitochondrial fractions. All fractions were stored at −80°C until use.

### Co-immunoprecipitation (co-IP) assay

For XAF1-Bax binding or Puma-Bax binding assay, cells were treated with Dex for 24 h. Cells (5×10^6^ cells/sample) were then harvested and lysed in RIPA buffer (Elpis Biotech) containing a protease inhibitor cocktail (Sigma-Aldrich). To reduce non-specific binding of protein, we performed pre-clearing on equal amounts of cell lysates by incubating samples with washed protein G PLUS-agarose beads (Santa Cruz Biotechnology). For IP, pre-cleared lysate plus the optimal amount of anti-XAF1 or -Puma antibody was incubated at 4°C for 2 h on a rotator. The immunoprecipitates were harvested by protein G PLUS-agarose beads (Santa Cruz Biotechnology) and incubated at 4°C for 2 h under rotary agitation. When incubation time was over, supernatant was removed and the beads were washed in lysis buffer four times. Finally, immunoprecipitates were eluted by boiling the beads in SDS-PAGE sample buffer for 5 min and then characterized by western blot analysis with appropriate antibodies.

## Results

### Dex induces apoptosis in EBV-transformed B cells but not in normal PBMCs

**Figure 1.**
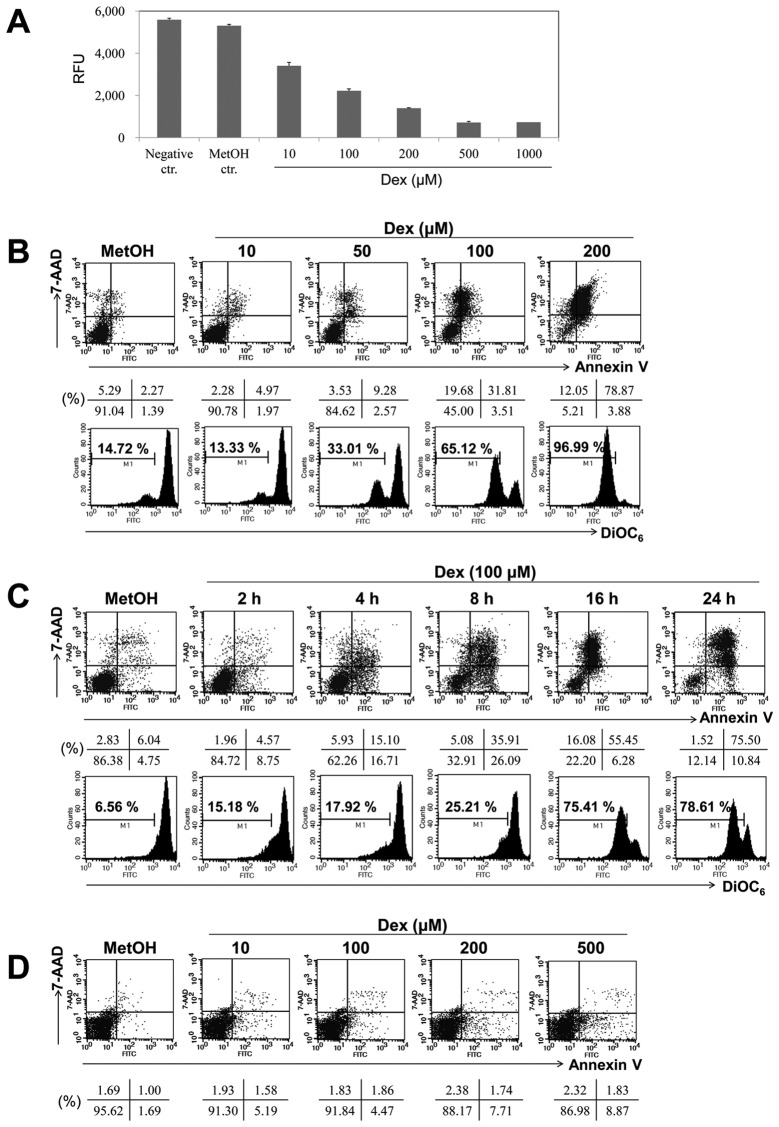
Dex induced apoptosis in a dose- and time-dependent manner in EBV-transformed B cells. (A) EBV-transformed B cells (5×10^4^ cells/well) were cultured in 96-well plates. After 24 h, cell proliferation was measured by AlamarBlue assay. RFU is the relative fluorescence unit. (B and C) EBV-transformed B cells and (D) PBMCs were treated with 10, 50, 100 and 200 *μ*M of Dex for 2, 4, 8, 16 and 24 h. The percentage of apoptotic cells was estimated by Annexin V/7-AAD staining. Dot plot graphs show percentage of viable cells (Annexin V^−^/7-AAD^−^), early-phage apoptotic cells (Annexin-V^+^/7-AAD^−^), late-phage apoptotic cells (Annexin-V^+^/7-AAD^+^), and necrotic cells (Annexin-V^−^/7-AAD^+^). To measure disruption of Δψ_m_, cells were stained with DiOC_6_. Diminished DiOC_6_ fluorescence indicates Δψ_m_ disruption and percentages indicates the cell proportion in each bar. Results are representative of three independent experiments.

We measured the effect of Dex on the proliferation of EBV-transformed B cells. Cells were treated with various doses of Dex (10, 100, 200, 500 and 1,000 *μ*M) for 24 h, and its effect on cell proliferation was analyzed by AlamarBlue assay. In the presence of Dex, EBV-transformed B cell proliferation was reduced in a dose-dependent manner, suggesting potential antitumor activity (Fig. 1A). Dex exhibited approximately 50% proliferation inhibition at dose of 100 *μ*M. To characterize the apoptosis response to Dex treatment in EBV-transformed B cells, Annexin V/7-AAD staining was performed. The cells were treated with different doses of Dex (0, 10, 50, 100 and 200 *μ*M) for 16 h. Fig. 1B shows that different doses of Dex has apoptotic-inducing effect on cells and that Annexin V and 7-AAD positive cells in MetOH-treated cells was 2.27%, whereas in treated cells with 10, 50, 100 and 200 *μ*M of Dex these were 4.97, 9.28, 31.81 and 78.87% after 16-h treatment, respectively. As shown in Fig. 1C, cells were exposed to Dex for diverse time intervals and Annexin V and 7-AAD positive cells in treated cells with Dex for 2, 4, 8, 16 and 24 h were 4.57, 15.10, 35.91, 55.45 and 75.50%. Moreover, Dex markedly induced Δψ_m_ dissipation (Fig. 1B, lower panel), especially between 8- and 16-h treatment (Fig. 1C, lower panel; from 25.21 to 75.41%). Because the optimal dose and time of Dex treatment were 100 *μ*M and 24 h, we chose this condition to evaluate protein alterations in Dex-induced apoptosis. Furthermore, we investigated whether Dex has any cytotoxic effect on normal human PBMCs and did not observed significant cell death in normal human PBMCs after Dex treatment (Fig. 1D). Our data suggest that Dex more selectively induces the cytotoxic effect on cancerous EBV-transformed B cells than normal human PBMCs.

### Dex induces caspase-dependent apoptosis in EBV-transformed B cells

**Figure 2.**
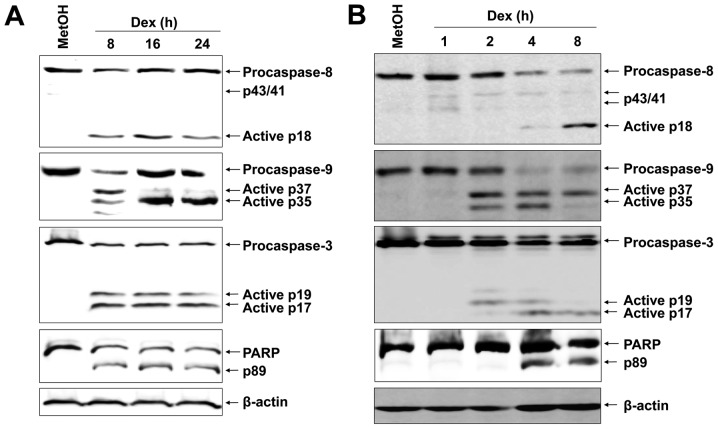
Effect of Dex on caspase activation. (A and B) EBV-transformed B cells were treated with 100 *μ*M Dex for the indicated times. Western blots of active caspase-8, -9, -3 and PARP cleavage were performed to characterize the apoptotic response. β-actin was used to normalized proteins contents. Results are representative of three independent experiments.

Based on the preliminary assays where a strong apoptotic effect of Dex was elicited in EBV-transformed B cells, the possible molecular mechanism underlying Dex-induced apoptosis was scrutinized. We first examined proteolytic processing of caspases by western blot analysis because activation of caspases has been reported to play a role in apoptosis mediated by various stimuli. Dex-induced apoptosis of EBV-transformed B cells were involved with activation of caspase-8, -9, -3 and PARP, as assessed by the appearance of the respective cleaved active caspases (Fig. 2A). Further, to elucidate the mechanistic order of caspases and PARP, we carried out short-term treatment with Dex. Interestingly, cleavage of procaspase-9 was detected as early as 2 h after Dex treatment (Fig. 2B). Cleavage of procaspase-8 into the characteristic 18-kDa active fragments was apparent 8 h after Dex treatment. We then examined effector caspase-3 and its substrate, PARP, and they were already increased at 4 h after Dex treatment and the amount dramatically increased at 8 h (Fig. 2B).

### Caspase-9 inhibition blocks activation of caspase-8

**Figure 3.**
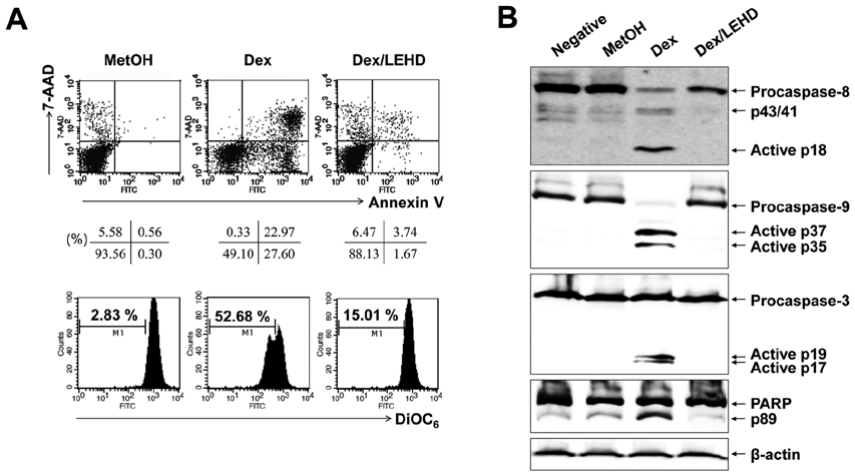
Caspase-9 is upstream caspase activated by Dex in EBV-transformed B cells. EBV-transformed B cells were pre-incubated with z-LEHD-fmk (20 *μ*M) for 2 h and then treated with 100 *μ*M Dex for 24 h. (A) The number of apoptotic cells (Annexin V/7-AAD) and Δψ_m_ (DiOC_6_) were obtained as described in Materials and methods. Percentages indicate the cell proportion in each quadrant and bar. (B) The cells lysate were subjected to western blot analysis with Ab against caspase-3, -8, -9 and PARP. Results are representative of three independent experiments.

The activation of caspase-8 is an initial caspase and could be due to direct activation of the death-receptor pathway. However, it could also be secondary signal to caspase-9/-3-linked cleavage, as reported in PUVA-induced apoptosis of T leukemia cells (20). Our experiments had shown that no FasL surface expression increased after Dex treatment of EBV-transformed B cells (data not shown). However, to survey this possibility further, we used selective caspase inhibitors. As depicted in Fig. 3, when EBV-transformed B cells had been pre-treated with a caspase-9 inhibitor (z-LEHD-fmk), this inhibitor blocked Dex-induced apoptosis and no cleavage of caspase-8 could be detected by western blot analysis performed on cells 24 h after Dex treatment. These results pointed to the likelihood that, in our system, activation of caspase-8 is secondary to the activation of caspase-9.

### Dex induces mitochondrial events related to apoptosis in EBV-transformed B cells

**Figure 4.**
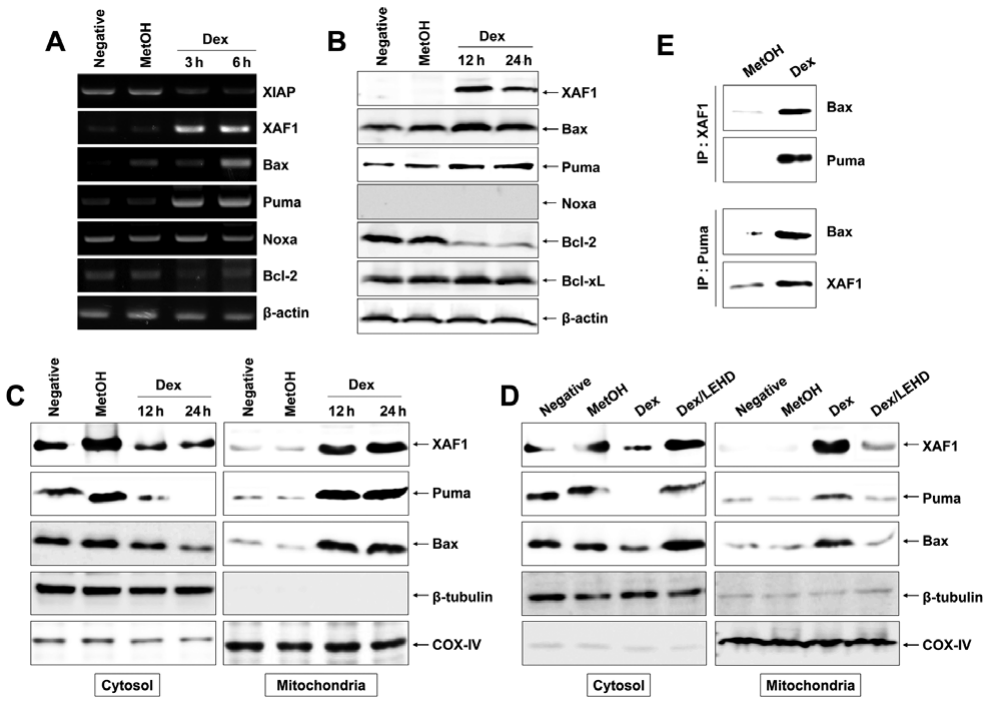
Activation of mitochondrial events in Dex-induced apoptosis in EBV-transformed B cells. Cells were treated with 100 *μ*M Dex for the indicated times. Total RNA and proteins were extracted from cell lysates and (A) RT-PCR for XIAP, XAF1, Bax, Puma, Noxa, Bcl-2 and β-actin mRNA and (B) western blot analysis for XAF1, Bax, Puma, Noxa, Bcl-2 and Bcl-xL protein were performed. (C and D) Cells were harvested and then the amount of Bax, XAF1 and Puma in cytosol and mitochondria fractions was determined. The mitochondria marker, COX-IV, and cytosol marker, β-tubulin were used to verify the purity of each fraction performed as described in Materials and methods. To block activation of caspase-9, cells were pretreated with z-LEHD-fmk (20 *μ*M) for 2 h. (E) In binding assay, XAF1 or Puma was immunoprecipitated using specific Ab, followed by immunodetection of Bax in immunoprecipitate as detailed in Materials and methods. Results are representative of three independent experiments. IP, immunoprecipitation; IB, immunoblot analysis.

Mitochondria-related proteins include anti-apoptotic proteins (Bcl-2, Bcl-xL, XIAP and survivin) and pro-apoptotic proteins (Bax, Bad, Bid, Puma, Noxa and XAF1). They can inhibit or activate the release of downstream factors which lead to the activation of caspase-3 and PARP in the execution of apoptosis. To investigate the apoptosis-related proteins, anti-apoptotic (Bcl-2, Bcl-xL and XIAP) and pro-apoptotic (Bax, Puma, Noxa and XAF1) were detected by RT-PCR and western blot analysis after cells were treated for different times with 100 *μ*M Dex. We found that Dex diminished the expression of XIAP, Bcl-2 and Bcl-xL mRNA, and protein levels. In contrast, Dex significantly increased the expression of XAF1 and slightly increased expression of Puma and Bax but had little effect on Noxa (Fig. 4A and B). It has been reported that Bax translocation promotes the rupture of mitochondrial outer membrane and facilitates the disruption of Δψ_m_(19). We separated the mitochondrial and cytosolic fractions after 12 and 24 h of Dex exposure to determine the Bax level by western blot analysis. As shown in Fig. 4C, there was a significant enhancement in translocation of Bax from cytosol to mitochondria at 12 and 24 h compared with control. In general, under normal environments, XAF1 was localized fundamentally in the nuclear (12), whereas XAF1 relocalization from nuclear to cytoplasm and mitochondria was observed after Dex exposure (Fig. 4C). Bax is required for XAF1 or Puma-mediated apoptosis and is translocated by XAF1 or Puma activation (19). To investigate whether XAF1 and Puma can interact with Bax directly, the interplay between XAF1 or Puma and Bax was performed using co-IP tool. The results of co-IP analysis indicate that the amount of Bax binding to XAF1 or Puma enhanced strongly after Dex exposure (Fig. 4C) and that the amount of XAF1 binding to Puma also increased after Dex exposure, suggesting that XAF1 and Puma have a parallel action and activate Bax. In addition, z-LEHD-fmk impeded translocation of XAF1, Puma, and Bax to the mitochondria (Fig. 4D), indicating that caspase-9 activation may precede translocation of XAF1, Puma and Bax.

### Dex leads to a persistent ERK1/2 phosphorylation in EBV-transformed B cells

**Figure 5.**
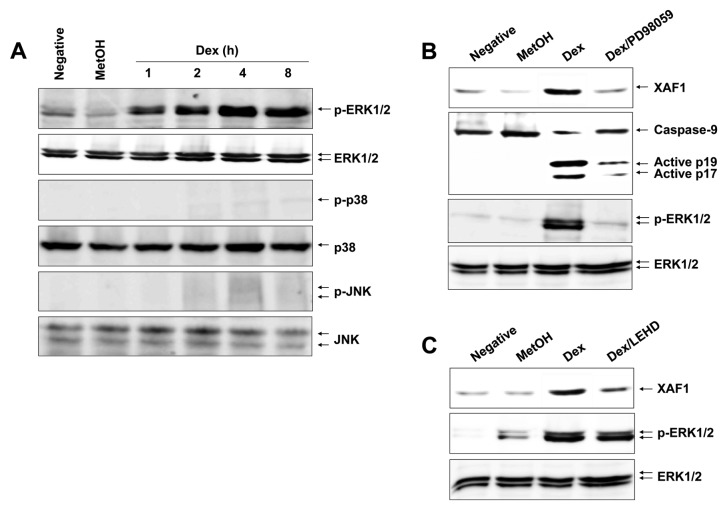
Dex causes a rapid activation of ERK1/2 in EBV-transformed B cells. (A) Cells were treated with 100 *μ*M Dex for the indicated time periods prior to cell lysis. Total protein was subjected to western blot analysis and successively immunoblotted against MAPKs. (B) Effect of ERK1/2 inhibitor on Dex-induced caspase-9 activation and XAF1 expression. To inhibit ERK1/2 phosphorylation, cells were pretreated with PD98059 (10 *μ*M) for 1 h. (C) Effect of caspase-9 inhibitor on Dex-induced ERK1/2 phosphorylation and XAF1 expression. To inhibit caspase-9 activation, cells were pretreated with z-LEHD-fmk (20 *μ*M) for 2 h. Results are representative of three independent experiments.

MAPK signaling associated with various cellular stresses and stimuli contributes to induction of apoptosis (21). We therefore tested whether certain MAPKs could induce expression and translocation of XAF1, Puma and Bax after Dex treatment. Cells were treated with Dex and analyzed for various MAPK activities, including ERK1/2, JNK and p38 MAPK. Fig. 5A shows that Dex apparently induced an activation of ERK1/2 after 1 h and a persistent phosphorylation level was observed up to 8 h in a time-dependent manner, whereas it had no effect on JNK and p38 MAPK. These results indicate that ERK1/2 is the potential inducer of Dex-induced XAF1 expression and trans-location. To corroborate the role of ERK1/2 in Dex-induced apoptosis, cells were treated with Dex in the presence or absence of the ERK1/2 inhibitor PD98059. The results show that the PD98059 inhibited Dex-induced XAF1 expression and attenuated caspase-9 activation (Fig. 5B). Moreover, caspase-9 inhibitor z-LEHD-fmk impeded XAF1 expression, whereas it did not block phosphorylation of ERK1/2 (Fig. 5C). Collectively, these observations substantiate the role of ERK1/2 in caspase-9 activation and XAF1 expression.

### ROS is critical for ERK1/2 activation and mitochondrial events by Dex

**Figure 6.**
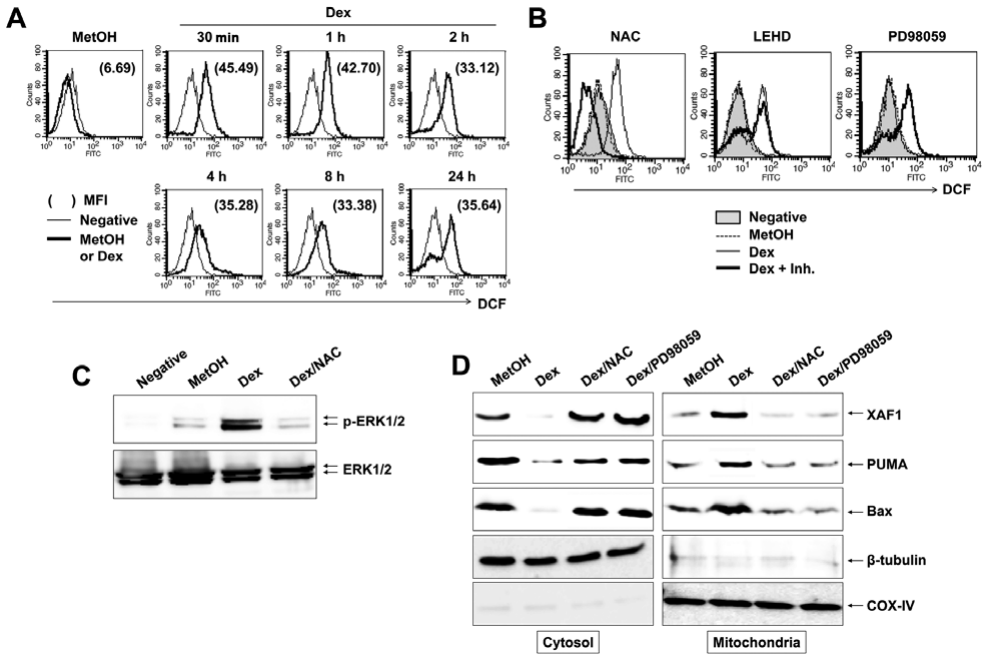
Dex induces ERK1/2 phosphorylation through ROS generation. (A) EBV-transformed B cells were pretreated with 10 *μ*M DCFH-DA for 30 min and then treated with 100 *μ*M Dex for the indicated time periods. MFI is the mean fluorescence intensity. (B) Effects of NAC, z-LEHD-fmk and PD98059 on Dex-induced ROS production. Cells were pretreated with the indicated inhibitors as described in Materials and methods before cells were treated with 100 *μ*M Dex or MetOH. The different inhibitors were applied at the following concentrations: NAC (10 mM), z-LEHD-fmk (20 *μ*M) and PD98059 (10 *μ*M). (C) Effect of ROS inhibitor on Dex-induced ERK1/2 phosphorylation. To inhibit the ROS production, cells were pretreated with NAC (10 mM) for 1 h. (D) Cells were harvested and then the amount of Bax, XAF1 and Puma in cytosol and mitochondria fractions were determined. To block ROS generation or ERK1/2 activation, cells were pretreated with NAC (10 mM) or PD98059 (10 *μ*M), respectively. Results are representative of three independent experiments.

In apoptosis, ROS is directly associated with activation of MAPKs and caspases (22). In addition, ROS is an early signal that provokes apoptosis (23) and a major source of action of many antineoplastic drugs. To establish whether reactive oxygen species (ROS) participate in Dex-induced apoptosis, cells were treated with 100 *μ*M Dex for the indicated times, followed by addition of DCFH-DA to measure intracellular ROS level. We found that Dex induced a marked increase in DCF fluorescence within 30 min and Dex-induced ROS level was maintained until 24 h (Fig. 6A). To elucidate the role of Dex-induced ROS generation on a persistent phosphorylation of ERK1/2 and caspase-9 activation during apoptosis, we treated cells with NAC, PD98059 or z-LEHD-fmk; inhibitors of ROS, ERK1/2 and casapse-9, respectively. Despite the inhibitors diminishing Dex-induced apoptosis remarkably (data not shown), as illustrated in Fig. 6B, NAC was the only inhibitor that significantly suppressed Dex-induced ROS production. Further, NAC inhibited phosphorylation of ERK1/2 by Dex treatment (Fig. 6C) and prevented translocation of XAF1/Puma/Bax complex to mitochondria (Fig. 6D). These findings strongly support that the critical role of ROS in Dex-induced persistent ERK1/2 phosphorylation.

## Discussion

Dex induces apoptosis and suppresses the mitogen-stimulated proliferation in various normal cells, including lymphoid cells (24) and fibroblasts (25). Therefore, it also represses cell growth of multiple myeloma (3), leukemia (4), prostate cancer (5), hepatoma (6), melanoma (7), osteosarcoma (8), lung cancer (9), breast cancer (10) and ovarian cancer cells (11). In the current study, we set out to elucidate the action mechanisms of Dex by which it induces apoptosis on EBV-transformed B cells. However, there is no information available concerning which proteins are the key inducers of apoptosis. We demonstrated, for the first time to our knowledge, that ROS generation and ERK1/2 activation induced XAF1 up-regulation in apoptosis.

Our results showed that Dex treatment of EBV-transformed B cells induced activation of caspase-9 and -8, as well as caspase-3. In time course experiments, our results suggested that caspase-9 was at the top of the hierarchy of the caspase cascade and is an initiator caspase to be evoked. This activation occured as early as 2 h after Dex treatment in EBV-transformed B cells, whereas caspase-3 and -8 showed activation after 4–8 h (Fig. 2). This postponement might agree with the formation of apoptosomes resultant from mitochondrial disruption (26), happening prior to adequate activation of the caspase signaling. Caspase-9 activation is consistent with the appearance of mitochondrial dysfunction reported by others in PUVA-treated Jurkat cells (27). The dramatic inhibition of Dex-induced apoptosis by z-LEHD-fmk strongly suggested that caspase-9 was the predominant upstream caspase in Dex-treated EBV-transformed B cell apoptosis (Fig. 3).

XAF1 is an essential mediator of apoptosis and plays a critical role in the induction of cell death (17,19). Previous reports have shown that the activation of JNK pathway was closely involved in XAF1-mediated apoptosis induction (28). Puma can be induced by DNA damaging drugs and is important in apoptosis (29). XAF1 or Puma induces mitochondria-mediated apoptosis by directly translocating Bax into mitochondria (29). Our current results indicate that Dex-induced apoptosis fulfills the molecular characteristics of the intrinsic pathway of apoptosis, including phosphatidylserine exposure, dissipation of Δψ_m_, and Bax, Puma and Bcl-2 conformational changes. More importantly, we found a significant increase both at the mRNA and protein level of the XAF1 during apoptosis. Our studies indicated that the expression of XAF1 was up-regulated and translocated into mitochondria in Dex-treated cells and XAF1 with Puma elicited translocation of Bax into mitochondria (Fig. 4). This suggested that XAF1 could be responsible for triggering the pro-apoptotic conformational changes of Bax. Immunoprecipitation further supported the main role of XAF1 in this apoptosis (Fig. 4E), suggesting that XAF1 is the most critical pathways through which Dex exerts an apoptotic effect in these cells.

The MAPK family proteins are mediators of diverse cellular reaction including proliferation, apoptosis and differentiation (30). They are composed of three protein kinases: ERK1/2, JNK and p38 MAPK (31). In general, transient activation of ERK1/2 takes part in the survival pathway (32). However, a previous report suggests that persistent activation of ERK1/2 contributes to cellular apoptosis in cervical cancer cells (33). Here we found that Dex treatment caused a persistent activation of ERK1/2 rather than JNK and p38 MAPK and persistent activation of ERK1/2 was involved in Dex-induced growth inhibition and apoptosis in EBV-transformed B cells (Fig. 5). Intriguingly, this was the first report that ERK1/2 functioned upstream of the XAF1 to induce cell apoptosis (Figs. 5B and 6D). PD98059, a specific inhibitor of ERK1/2, effectively reversed Dex-induced apoptosis and attenuated Dex-induced XAF1 expression, suggesting the pro-apoptotic effects of Dex in EBV-transformed B cells are mediated by a persistent activation of the ERK1/2 signaling pathway. Our study indicated that the ERK1/2-induced XAF1 with Puma promoted translocation of Bax into mitochondria in Dex-exposed cells (Fig. 6D).

Oxidative stress can be elicited by ROS, which refers to persistent excessive ROS production and limited antioxidant shield, and has been involved in various biological responses such as apoptosis (22,23). Accumulating evidence indicates that chemical-mediated ROS production gives rise to alteration of cellular function and eventually results in apoptosis. Dysfunction of mitochondria, induced by the production of excessive ROS, leads to dissipation of Δψ_m_ and apoptosis (23). Moreover, ROS signaling appears to be triggered by the activation of the mitochondrial-dependent cell death pathway through activation of MAPK pathways (22). Our data indicated that a persistent phosphorylation of ERK1/2 was caused by ROS generation after Dex treatment. Thus, Dex was shown to induce a boost of ROS with a peak after a 30 min exposure, and a pretreatment with the ROS scavenger NAC significantly decreased the persistent ERK1/2 phosphorylation. We found that ERK1/2 was involved in this apoptotic effect of Dex in a ROS-dependent manner (Fig. 5). Inhibition of ERK1/2 reversed Dex-mediated apoptosis. Dex induced apoptosis of EBV-transformed B cells by ROS-dependent ERK1/2-mediated XAF1 up-regulation (Fig. 6).

In conclusion, we found that Dex inhibited cell growth and induced apoptosis in EBV-transformed B cells. Our results indicated that Dex-induced apoptosis was involved in the reduction of XIAP, Bcl-xL and Bcl-2 expression and induction of Bax, Puma and XAF1 expression, and caused the dissipation of Δψ_m_ in EBV-transformed B cells. Our results also demonstrated that Dex induced the activation of caspase-9 as initiator caspase and subsequently induced the activation of caspase-3 and -8. More importantly, ROS, ERK1/2 and XAF1 participated in Dex-induced apoptosis. Therefore, we demonstrate that Dex mediates apoptosis of EBV-transformed B cells through a novel ROS-dependent ERK1/2-mediated XAF1 signaling pathway.

## Abbreviations:

EBV: Epstein-Barr virus;
Dex: dexamethasone;
ROS: reactive oxygen species;
NAC: N-acetyl-l-cysteine
